# The links between neuroinflammation, brain structure and depressive disorder: A cross-sectional study protocol

**DOI:** 10.1371/journal.pone.0311218

**Published:** 2024-11-20

**Authors:** Egle Milasauskiene, Julius Burkauskas, Simonas Jesmanas, Rymante Gleizniene, Vilmante Borutaite, Kristina Skemiene, Paulina Vaitkiene, Virginija Adomaitiene, Saulius Lukosevicius, Brigita Gradauskiene, Guy Brown, Vesta Steibliene

**Affiliations:** 1 Laboratory of Behavioral Medicine, Neuroscience Institute, Lithuanian University of Health Sciences, Kaunas, Lithuania; 2 Department of Radiology, Lithuanian University of Health Sciences, Kaunas, Lithuania; 3 Laboratory of Biochemistry, Neuroscience Institute, Lithuanian University of Health Sciences, Kaunas, Lithuania; 4 Laboratory of Molecular Neurobiology, Neuroscience Institute, Lithuanian University of Health Sciences, Kaunas, Lithuania; 5 Psychiatry Clinic, Lithuanian University of Health Sciences, Kaunas, Lithuania; 6 Department of Immunology and Allergology, Lithuanian University of Health Sciences, Kaunas, Lithuania; 7 Department of Biochemistry, University of Cambridge, Cambridge, United Kingdom; Nathan S Kline Institute, UNITED STATES OF AMERICA

## Abstract

**Introduction:**

It is known that symptoms of major depressive disorder (MDD) are associated with neurodegeneration, that lipopolysaccharide (LPS) can induce symptoms of MDD, and that blood LPS levels are elevated in neurodegeneration. However, it is not known whether blood LPS and cytokine levels correlate with MDD, cognition and brain structure, and this is tested in this study.

**Methods and analysis:**

This cross-sectional study includes individuals with MDD (*n* = 100) and a control group of individuals with no one-year history of a mental disorder (*n* = 50). A comprehensive evaluation is performed, including the collection of basic sociodemographic information, data on smoking status, body mass index, course of MDD, past treatment, comorbid diseases, and current use of medications. Diagnosis of MDD is performed according to the WHO’s [2019] International Classification of Diseases and related health problems by psychiatrist and severity of MDD is evaluated using the Montgomery–Åsberg Depression Scale. The Cambridge Neuropsychological Test Automated Battery is used to evaluate cognitive functioning. Venous blood samples are taken to measure genetic and inflammatory markers, and multiparametric brain magnetic resonance imaging is performed to evaluate for blood-brain barrier permeability, structural and neurometabolic brain changes. Descriptive and inferential statistics, including linear and logistic regression, will be used to analyse relationships between blood plasma LPS and inflammatory cytokine concentrations in MDD patients and controls. The proposed sample sizes are suitable for identifying significant differences between the groups, according to a power analysis.

**Administrative information:**

**Trial registration:** Clinicaltrials.gov NCT06203015.

## Introduction

Major depressive disorder (MDD) is a chronic mental illness, with a 60% lifetime risk of recurrence after the first MDD episode [1]. It begins at an early age; has a negative impact on a person’s work, social, and domestic functioning; and is associated with poorer health-related quality of life [2]. The Global Burden of Diseases, Injuries, and Risk Factors 2019 Study revealed that MDD ranked among the top 25 leading causes of global disease burden in 2019 [3]. Even more concerning is the fact that there has been no reduction in the global prevalence or burden of MDD since 1990s [4], despite compelling evidence of interventions that could mitigate its impact. Additionally, the Covid-19 pandemic has had a detrimental effect on the prevalence and burden of MDD [5]. It is projected that by 2030, MDD will become the leading cause of disease burden worldwide [6]. Despite available treatment options for MDD, only about half to two-thirds of patients respond to first-line antidepressant treatment, and only 30% to 45% of patients achieve remission [7]. This low remission rate and high rate of treatment resistance may be due to the polyetiological nature of the disease, the heterogeneity of the clinical picture of depression, and the lack of biomarkers to stratify MDD subtypes [8, 9]. Inadequate treatment efficacy for MDD also has a negative impact on the efficacy, duration, outcomes, and survival rates of treatment for comorbid somatic disorders [10, 11]. A recent review by the World Psychiatric Association (WHO) Commission described depression and its associated consequences as potentially avoidable and highlighted the heterogeneity of the disease and the need for personalised treatment strategies and further research of pathophysiological mechanisms [12].

The aetiology of MDD, although researched extensively, remains unclear. In the 1950s, the monoamine hypothesis of depression was formulated [13], suggesting that the underlying pathophysiological basis of depression is a depletion of serotonin, norepinephrine, and/or dopamine levels in the central nervous system. This hypothesis has been influential for decades and provided an important justification for the prescription of antidepressants. As the rates of treatment-resistant cases and residual symptoms increased, the hypothesis started to be questioned, and a recent systematic review found insufficient evidence of link between serotonin and depression [14]. Other known factors affecting depression include abnormalities in the hypothalamic-pituitary-adrenal axis and thyroid hormones, genetic factors, environmental stress factors, reduced neurogenesis, increased cytokines secretion, neuroinflammation and neurodegeneration. Nevertheless, none of these proposed mechanisms alone explains the pathogenesis of depression.

Over the past two decades, accumulated scientific evidence has supported the importance of the immune system in the etiopathogenesis of MDD [15–19]. The activation of the immune system and the effects of pro-inflammatory and anti-inflammatory cytokines are emphasised in most of the main theories of the pathophysiological mechanisms of MDD [20–23]. Various studies have reported higher concentrations of multiple inflammatory markers, including interleukin [IL]-6, IL-10, tumor necrosis factor alpha (TNFα) and interferon gamma (INFγ) in patients with MDD [24]. However, these findings were noted in only a subgroup of patients with depression. This subtype of depression, so-called *inflammatory depression* or *inflamed phenotype* has been associated with the recurrence of depressive episodes [25], treatment resistance [26], illness severity [27] and a specific symptom profile, including changes in appetite and sleep, fatigue and cognitive dysfunction [28–30].

Further evidence for a role of inflammation in depression comes from treatment studies. The administration of several classes of compounds with anti-inflammatory effects suggests that these drugs may have antidepressant effects, but results differ between studies, with some authors suggesting that the variability of effects may be due to heterogeneity in the inflammatory alterations, or to the absence of validated stratification algorithms incorporating several peripheral markers of inflammation, and noting that none of the markers is convincingly predictive of neuroinflammation amongst patients with MDD [31–36]. In addition, only a few studies have examined the relationship between inflammatory markers and depression with stratification of groups according to smoking status and body mass index, which are known to be sources of peripheral inflammation.

Cognitive dysfunction is a common residual symptom in patients with response to selective serotonin reuptake inhibitors whose MDD has gone into remission [37, 38], and is a principal determinant of functional nonrecovery. The affected functions included executive functioning, memory, and attention [37, 39]. Unipolar depression has been associated with an increased risk of developing dementia [40]. Levels of inflammatory markers have been found to be associated with cognitive performance in MDD. Higher levels of IL-6 and CRP were negatively associated with executive function, attention, verbal memory and psychomotor speed [41–43]. CRP and IL-6 levels are not only associated with cognitive symptoms of depression at baseline but also predicted those symptoms at a 12-year follow-up [28].

Until now, the cause of the low-grade inflammation observed in this subgroup of MDD patients has been unclear. In the proposed study, we will test a new hypothesis of the origin and pathophysiology of MDD: the endotoxin hypothesis of depression. This hypothesis states that endotoxin, a lipopolysaccharide (LPS) that constitutes much of the outer membrane of gram-negative bacteria and is present at high concentrations in the gut, causes MDD or inflammatory depression specifically. Blood plasma levels of LPS are normally low but are elevated during infections, gut inflammation, gum disease, and neurodegenerative diseases [44, 45]. Dysbiosis may promote increased intestinal permeability (“leaky gut”), which leads to bacterial translocation across the intestinal barrier and into the circulation, thus increasing blood plasma levels of LPS, which triggers the secretion of cytokines. Data suggest that plasma LPS or LPS-induced peripheral inflammation can induce inflammation in the brain (neuroinflammation) and symptoms of MDD [46–49]. Thus, gut dysfunction or infections could elevate plasma LPS that causes inflammatory depression. However, it is not known whether plasma LPS levels are elevated in MDD or inflammatory depression, or associated with specific symptoms of MDD. This is tested by the protocol outlined here.

Elevated plasma levels of LPS have been associated with neurodegeneration, and LPS can induce neurodegeneration, leading to the endotoxin hypothesis of depression, i.e. that endotoxin causes or contributes to neurodegeneration [44]. This protocol tests this hypothesis, and in particular whether elevated plasma levels of LPS are associated with cognitive dysfunction, permeability of the blood-brain barrier, brain vascular or white matter damage or other changes in brain structure associated with neurodegeneration.

Data from preclinical studies show that LPS not only causes an inflammatory process in the central nervous system but also inhibits hippocampal neurogenesis [50]. Most previous clinical studies of brain changes related to systemic inflammation have focused on single or several brain magnetic resonance imaging (MRI) parameters and their associations with systemic inflammatory markers [51–53]. For example, one study showed that peripheral inflammation may contribute to impaired microstructural integrity of corticolimbic white matter pathways [51]. Another showed that higher levels of pro-inflammatory cytokines are associated with higher levels of glutamate in the anterior cingulate cortex among patients with MDD [52]. There remains a need for multiparametric brain MRI studies to better understand various possibly interlinked etiopathological processes involved in depression and inflammation that may manifest as changes in volume, white matter microstructural integrity, perfusion, and neurometabolism. In particular, the effect of regional blood-brain barrier (BBB) disfunction and how this relates to depression and inflammation deserves more attention in the clinical literature, because the BBB is a crucial structure in the interplay of systemic inflammation and changes in the brain environment [54]. Based on existing preclinical and clinical research data, we hypothesise that an increase in blood plasma LPS and peripheral cytokines induce BBB dysfunction, neuroinflammation and neurodegenerative processes in specific etiologically relevant structures of the brain and cause clinical manifestation of depressive symptoms and cognitive damage. It is known that depressive symptoms can be an early expression of neurodegeneration, but a relationship between the level of LPS and the manifestation of MDD has not yet been tested.

Also, MDD is a multifactorial mental disorder, with genetic factors contributing to an individual’s susceptibility to its development. While no single gene is solely responsible for MDD, existing evidence indicates that certain genes and genetic variations, implicated in neurotransmitter regulation, initiation and progression of inflammatory processes, neuroplasticity, and the stress response, may heighten an individual’s vulnerability to MDD. Based on the literature data, in this study we are going to investigate the effects of single nucleotide polymorphisms of four genes in relation to blood plasma LPS and peripheral cytokines concentrations and clinical manifestation of MDD: *Brain-derived neurotrophic factor (BDNF) rs6265* gene [55], *Glycogen synthase kinase 3β (GSK3β) rs6438552* gene [56], *MAPK1 rs6928*, *protein encoding gene* [57], and *synapsin 1 encoding gene -SYN1* [58].

In conducting this research, we have two primary aims, to:

1. Evaluate the concentrations of blood plasma LPS and inflammatory cytokines among patients with MDD and in a control group. We hypothesize that an increase in blood plasma LPS is associated with an increase in blood inflammatory cytokines in the group of patients with MDD.
2. Evaluate the severity and different symptoms of MDD, and relate these to blood plasma levels of LPS and inflammatory cytokines. We hypothesize that LPS and inflammatory markers are associated with MDD and/or specific symptoms of MDD, allowing us to identify MDD patients with a specific LPS-associated inflammatory subtype of depression.
  Secondary aims include the following:
3. Perform a multiparametric brain MRI assessment of depressed patients and a control group. We hypothesize that, among patients with MDD, there are changes in blood-brain barrier permeability to water, levels of neuroinflammation and perfusion, and structural neurodegenerative changes that are associated with blood plasma LPS and cytokine concentrations.
4. Evaluate cognitive functions. We hypothesize that low-grade neuroinflammation negatively affects cognitive performance among patients with MDD.
5. Perform a subgroup analysis on neuroinflammation markers based on individuals’ BMI and smoking status.
6. Determine possible genetic markers among MDD patients in relation to blood plasma LPS and inflammatory cytokines concentrations that could predict a genetic predisposition to neuroinflammation, neurodegeneration and the development of MDD.

## Methods and analysis

### Participants

Two groups of subjects are invited to participate in this cross-sectional study: (1) patients diagnosed with MDD (according to the WHO’s [2019] International Classification of Diseases and related health problems [59] categorization of mental disorders, diagnosed by a psychiatrist) and (2) a control group of subjects who have not been diagnosed with any mental disorder in the past year. All subjects will be ≥18 years old and will sign informed consent. Exclusion criteria for the MDD group are as follows: diagnosis of other mental disorders during the past one-year period, diagnosis of somatic diseases that may affect changes in inflammatory factors in the body. Exclusion criteria for the control group are as follows: diagnosis of any mental disorders within the past one-year period, previous suicide attempt, or current suicide risk identified in the study, and diagnosis of somatic diseases that may affect changes in inflammatory factors in the body.

### Recruitment

The study is being conducted at the hospital of the Lithuanian University of Health Sciences Kaunas Clinics Psychiatry Clinic (inpatient department), and the Nervous System Diseases Outpatient Department. The recruitment for the study has started in June 2022 and is still ongoing. Patients for the MDD group are being referred by a psychiatrist. An advertisement has been prepared to invite participants for the control group to take part in this research and is shared among primary care physicians.

### Overview of protocol

A one-day visit to the research centre is expected. All subjects undergo the same procedures:

1. Subjects complete a questionnaire prepared by the researchers, collecting medical history, sociodemographic data and information about smoking status, BMI, data on the course of MDD, past and current treatment.
2. The diagnosis of MDD is performed by doctor psychiatrist, according to the WHO’s [2019] International Classification of Diseases and related health problems [59]. Clinical expression of MDD symptoms is assessed by a psychiatrist using a standardized clinical instrument: the Montgomery-Åsberg Depression Rating Scale (MADRS).
3. The Cambridge Neuropsychological Test Automated Battery (CANTAB) is used to determine cognitive dysfunction [60]. Tests are performed by a clinical psychologist and a psychiatrist with clinical experience working with this instrument.
4. Venous blood samples are taken by a medical nurse to measure levels of cytokines, LPS, and genetic markers.
5. Multiparametric brain MRIs are performed by a radiology technologist and a radiologist, and analysed by two radiologists.

Study investigators and staff will provide feedback to all participants via a telephone call 7 days after the completion of the study, regarding the available results of completed procedures. Schedule of enrolment, interventions, and assessments is presented in S1 Table.

### Study measures

#### MADRS

The MADRS [61] is a clinician-rated questionnaire used to measure the severity of depressive episodes. This questionnaire consists of 10 items, with each item rated on a 0-to-6 Likert-type scale. The overall sum score ranges from 0 to 60, with higher scores indicative of greater depressive symptomatology. The Lithuanian version of the MADRS has been adapted in a sample of 522 patients with coronary artery disease, demonstrating a one-factor structure and high internal consistency [62]. At a cut score value of 10 or higher, the Lithuanian version of the questionnaire exhibits good psychometric properties for the identification of a current depression episode [62].

#### CANTAB

All study participants will complete tests from the CANTAB (2018) including the screening test (Motor screening), as well as tests assessing visual memory (Delayed Matching to Sample test), set-shifting (Intra-Extra Dimensional Set Shift), spatial planning (One Touch Stockings of Cambridge), working memory and strategy use (Spatial Working Memory task), attention (Match to Sample Visual Search and Rapid Visual Information Processing tasks), and decision making (Cambridge Gambling Task). These tests specifically measure cognitive impairment related to depression and have been validated in numerous clinical investigations [63–67].

#### Blood samples

Sixteen milliliters of peripheral venous blood will be drawn from each patient in the morning (after fasting for at least 8 hours). Blood will then be centrifuged to produce serum samples, which will be stored at −80°C for testing. Commercial ELISA kits will be used to measure levels of LPS and inflammatory cytokines (IL-6, IL-10, INF-γ and TNF-α).

#### Genetic testing

Venous blood samples for DNA extraction will be collected in ethylenediaminetetra-acetic acid tubes. Genomic DNA from peripheral blood leucocytes will be extracted using a genomic DNA purification kit (Catalog No. K0512; Thermo Fisher Scientific, Waltham, MA, USA) according to the manufacturer’s recommendations. Several polymorphisms in genes involved in several neurotrophic/neuroplasticity pathways will be evaluated, namely, *BDNF (rs6265)*, *GSK3B (rs6438552)*, *MAPK1 (rs6928)*, and *SYN1 (rs1142636)*, by using a commercially available genotyping kit (Applied Biosystems; Foster City, CA, USA). A 9-μl reaction volume will consist of 1 μl of genomic tumor DNA (20 ng/μl), 5 μl of TaqMan® Universal PCR Master Mix (Thermo Fisher Scientific), 0.2 μl of TaqMan® SNP Genotyping Assay, and 3.8 μl of H2O. Negative water control will be used in every assay. Samples will be incubated in a qPCR thermocycler for 10 min at 95°C, 40 cycles (15 sec at 92°C, 1 min at 60°C), 1 min at 60°C, and then held at 4°C. Finally, allelic discrimination will be performed using software provided by Applied Biosystems.

#### Brain MRI

Multiparametric brain MRI assessment will aim to evaluate BBB permeability to water, brain structure volumes, white matter integrity, cerebral perfusion, and neurometabolite concentrations. Brain MRI will be performed on a 3 Tesla Siemens Skyra scanner (Siemens Healthcare GmbH, Erlangen, Germany) with the following sequences: T1W/mpr/p2/iso and T2W_spc_dark-fluid (for volumetric and anatomical information), SWI (for detection of hemosiderin and calcifications), DTI (for analysis of white matter integrity), 3D GRASE diffusion-prepared pCASL (DP-pCASL) [68] (for evaluation of cerebral perfusion and BBB permeability to water), single voxel 1H-MRS in four locations: left amygdala, left dorsal anterior cingulate cortex, left dorsolateral prefrontal cortex, and left ventromedial prefrontal cortex (for neurometabolic analysis). Quantitative analysis of MRI data will be performed predominantly on open-source software, using FreeSurfer/TRACULA (Martinos Center for Biomedical Imaging), AFNI (Scientific and Statistical Computing Core of the National Institute of Mental Health Intramural Research Program), LCModel (Steven Provencher), while BBB permeability and perfusion analysis will be performed on proprietary software acquired from the DP-pCASL sequence creators at the University of Southern California’s Stevens Neuroimaging and Informatics Institute [68, 69].

#### Statistical analysis

The research sample was calculated based on standard sample size formulas [70], choosing 5% error and 95% probability (one-sided 5% test and 80% power), finding that 100 patients with MDD and 50 control group subjects are expected to present statistically significant conclusions based on previous research data. We will use SPSS (Version 29.0; IBM, Chicago, IL, USA), and Mplus (Version 8.0; Los Angeles, CA, USA) to run all statistical analyses. We will use multiple linear and logistic regression models, providing the unstandardised regression coefficient B and the odds ratio, respectively, with their 95% confidence intervals, to examine correlations between MDD and indicators of inflammation, cognitive function, and MRI.

#### Access to data

Study data sets will be password protected. Study Principal Investigator and Investigators will have direct access to data sets. To ensure confidentiality, data dispersed to study team members will be blinded of any identifying participant information.

### Ethics and dissemination

The approval from the Kaunas Regional Biomedical Research Ethics Committee (KRBREC) to conduct a study has been obtained (No. BE-2-11, 18.01.2022 and updated version No. P1-BE-2-11, 17.02.2023). All subjects will be informed about the study using a personal information form. Subjects will be included in the study if they agree to participate and sign the informed consent form. Trial investigator will obtain written consent from patients willing to participate in the trial. The study will be conducted in accordance with good clinical practice guidelines and the principles of the Declaration of Helsinki. During the study, confidential data related to each person`s health will be collected. The confidentiality of all data will be maintained within legal limits. Participants’ study information will not be released outside of the study without the written permission of the participant, except as necessary for monitoring by KRBREC. All the study data and blood samples will be destroyed 15 years after the entire study is finished and will not be used in future studies. Any modifications to the protocol which may impact on the conduct of the study, potential benefit of the patient or may affect patient safety, including changes of study objectives, study design, patient population, sample sizes, study procedures, or significant administrative aspects will require a formal amendment to the protocol. Such amendment will be approved by the KRBREC prior to implementation and notified to the health authorities in accordance with local regulations.

### Post-trial care

The interventional methods applied during this study may cause only minor unwanted temporary effects on participants’ health. If participants suffer material and non-material damage to health, as a result of participating in the study, it would be compensated in accordance with the procedure established by the Law on Patient Rights and Compensation for Health Damage from the State health funds under Ministry of Health of the Republic of Lithuania.

### The patient and public involvement statement

The KRBREC, inclusive of a patient and public representative, has ethically reviewed and endorsed the study, considering intervention burden, time commitment, and result dissemination. The approved dissemination plan comprises presentations to MDD-focused patient organizations, addressing etiology, pathogenesis, neuroinflammation’s role, the need for further endotoxin-associated MDD research, and potential treatment perspectives. Study results will be communicated through online Lithuanian newspapers, general public social media platforms, and media interviews. These communication items are planned on Suicide Prevention Day and during Open Psychiatry Month in Lithuania.

### Dissemination policy

Study results will be published in open-access medical journals and presented at international and national medical conferences for healthcare professionals. Medical writers are not intended to be used in this study. When publishing the results of the studies, relevant data will be provided as supporting information files or will be made available by the authors, without undue reservation.

## Discussion

For all the reasons listed earlier, the issue of MDD treatment effectiveness is of great importance. Current evidence supports the assumption that inflammatory factors contribute to the onset of MDD, the maintenance of depressive symptoms, and the recurrence of depressive episodes, making them a potential therapeutic target for a subgroup of MDD patients. Thus, there is an urgent need to identify immune markers and clinical phenotypes of an inflammatory subtype of MDD. Our study will test whether elevated plasma LPS is associated with MDD, an inflammatory subtype of MDD, specific symptoms of MDD or cognitive deficits. The results of our study could help identify a group of patients with LPS associated inflammatory depression whose pathogenesis involves changes in the immune system related to LPS and inflammatory cytokines. Additionally, this study could reveal a relationship among regional blood-brain barrier disruption, LPS-associated low-grade neuroinflammation, and neurodegenerative brain changes, particularly in frontolimbic regions and the hippocampus. This could, in turn, clarify the etiopathogenetic basis of cognitive damage commonly observed in both depressed and remitted patients. To the best of our knowledge, such results would be the first of their kind in the world as of the time of writing this article.

While an extensive literature already exists on the genetic factors associated with MDD, there is much less reported in relation to the differences between individuals with different pathogenetic mechanisms of MDD. In this study, we are going to investigate the possible effects of single nucleotide polymorphisms of four genes known to be associated with several neurotrophic/neuroplasticity pathways relevant to the mechanism of action of antidepressants. BDNF is crucial for neuronal survival and growth, acts as a neurotransmitter modulator, and is involved in neuronal plasticity, which is vital for learning and memory [71]. Several lines of evidence suggest that the *BDNF rs6265* polymorphism could be involved in depression [58, 72, 73]. GSK3β, initially described as a negative regulator of glycogen synthesis, is a molecular hub linking numerous signaling pathways in a cell, possibly linked to symptoms of depression [56, 74]. *MAPK1* is a key signaling kinase, which plays a critical role in synaptic and structural plasticity. *MAPK1* is also involved in the initiation and progression of inflammatory processes, highly related to depressive states [57]. The *SYN1* gene encodes synapsin I, a neuronal phosphoprotein associated with the membranes of small synaptic vesicles. Synapsins may play a role in synaptic neurotransmission, neuronal development, synaptogenesis, maintenance of mature synapses, and plasticity [75]. Findings suggest that the *SYN1* gene may play a role in the development of treatment-resistant depression [58]. The results of this study will reveal possible associations between these genetic polymorphisms in a cohort of Lithuanian patients and inflammatory markers, a specific depression symptom profile, and multiparametric brain MRI assessment data. These associations will provide new knowledge about possible molecular mechanisms of the disease.

In addition, the results of our study would provide an important pathophysiological basis for clinical trials targeting specific patients, potentially leading to the development of new individualized and clinically effective treatment strategies. Confirmation of LPS’s role in the etiopathogenesis of MDD would open new possibilities in the treatment and prevention of MDD, for example by reducing LPS levels or inhibiting LPS-contributed neuroinflammation. This could also lead to an increase in the efficiency of MDD treatment, a reduction in disease burden, and lower MDD-related suicide rates. Our results will offer opportunities for testing for neurocognitive biomarkers of depression related to low-grade neuroinflammation, which may complement clinical assessments in individuals with MDD.

The study may also identify whether elevated plasma LPS in MDD is associated with cognitive deficits or brain structural changes consistent with neurodegeneration. If this is confirmed, then reduction of LPS could become a target for preventing the neurodegenerative process.

The strengths of our study include well-validated scales and cutting-edge neurocognitive and biological assessments. Although this research will be carefully prepared, there are several limitations to be considered. A first and major limitation of this study is its cross-sectional design, which will not allow the differentiation of possible causal relationships between LPS-associated inflammatory depression and MDD. Thus, we will not collect any prospective observations that could contribute to an understanding of the formation of MDD psychopathology associated with neuroinflammation. Second, the generalizability of our results may be limited because this study will be conducted with a relatively small sample of individuals with MDD attending a single medical unit. Third, we acknowledge that several other factors might play a role in MDD formation due to neuroinflammation; however, we view this study protocol as a starting base for future studies to conduct a more comprehensive analysis of possible contributing factors. Fourth, we will not include medication in our statistical analysis because we assume these individuals will be receiving standard naturalistic treatment for MDD. Nevertheless, it is important to note that the effects of certain medications may influence an individual’s neurocognitive performance.

In summary, drawing from available preclinical and clinical research findings, we will investigate, for the first time, novel hypotheses that posit correlations between elevated blood levels of LPS, peripheral cytokines, disruption of the BBB, neuroinflammation, neurodegeneration in specific brain structures, MDD, MDD symptoms and cognitive impairment in clinical settings. Furthermore, we may identify a new subtype of MDD–LPS-associated inflammatory depression ‐ with a specific pathogenetic mechanism and clinical presentation.

## Supporting information

S1 Table Schedule of enrolment, interventions, and assessments. (DOCX)

S1 Checklist SPIRIT 2013 checklist: Recommended items to address in a clinical trial protocol and related documents*. (DOC)
