# Supplementary material for: The links between neuroinflammation, brain structure and depressive disorder: A cross-sectional study protocol
Source: PLoS One. 2024 Nov 20;19(11):e0311218. doi: 10.1371/journal.pone.0311218 (PMC11578540; doi:10.1371/journal.pone.0311218)
Supplement: S1 Table — (DOCX) [file pone.0311218.s001.docx]

**S1 Table. Schedule of enrolment, interventions, and assessments.**

|  | **Study period** | | |
| --- | --- | --- | --- |
| **Event** | Day -7 to 0 | Day 1 | Day 7 |
| Signed/confirm ICF | X |  |  |
| Assessment of eligibility | X |  |  |
| Medical Hx/Demographics |  | X |  |
| MADRS |  | X |  |
| CANTAB |  | X |  |
| Blood samples |  | X |  |
| Brain MRI |  | X |  |
| Results of the assessments |  |  | X |

Cambridge Neuropsychological Test Automated Battery, **CANTAB**; Magnetic resonance imaging, **MRI**; the Montgomery-Åsberg Depression Rating Scale, **MADRS**; Informed consent form, **ICF**.
